# Surgical Outcomes of the Non‐renorrhaphy Technique During Robot‐Assisted Partial Nephrectomy for Highly Complex Renal Tumors (RENAL Score ≥ 10)

**DOI:** 10.1111/iju.70157

**Published:** 2025-06-24

**Authors:** Kosuke Hamada, Takeshi Yamasaki, Shoma Yamamoto, Taisuke Matsue, Nao Yukimatsu, Taiyo Otoshi, Minoru Kato, Katsuyuki Kuratsukuri, Junji Uchida

**Affiliations:** ^1^ Department of Urology Graduate School of Medicine, Osaka Metropolitan University Osaka Japan

**Keywords:** complex, nonrenorrhaphy, partial nephrectomy, renal tumors, robot‐assisted

## Abstract

**Objectives:**

Non‐renorrhaphy technique during partial nephrectomy has not been sufficiently studied. This study aimed to evaluate the surgical outcomes of the non‐renorrhaphy technique in robot‐assisted partial nephrectomy (RAPN) for tumors with RENAL scores of 10–12.

**Methods:**

We retrospectively analyzed 51 consecutive patients who underwent RAPN with or without renorrhaphy for RENAL score 10–12 tumors at Osaka Metropolitan University Hospital between March 2015 and December 2023. Perioperative outcomes were compared between 16 patients in the nonrenorrhaphy group and 35 patients in the renorrhaphy group. Univariate and multivariate linear regression analyses were conducted to identify predictors of renal function and renal parenchymal volume preservation.

**Results:**

Patient demographics and preoperative tumor characteristics exhibited no significant differences between the two groups. Operative time was significantly shorter in the nonrenorrhaphy group (185 vs. 217 min, *p* = 0.0016). The preservation rate of renal parenchymal volume was significantly higher in the nonrenorrhaphy group (86.7 vs. 74.2%, *p* = 0.0016), but there was no significant difference in the preservation rate of estimated glomerular filtration rate (*p* = 0.6380). No significant difference was observed in the incidence of major complications (Clavien‐Dindo grade ≥ 3) between the two groups. Urinary leakage occurred in both groups (*p* = 0.9399). In univariate and multivariate analyses, renorrhaphy and clinical tumor size were identified as significant predictors of renal parenchymal volume preservation.

**Conclusions:**

Even in cases with RENAL scores of 10–12, the non‐renorrhaphy technique appears to be a feasible and safe approach, and may be associated with better preservation of renal parenchymal volume.

**Abbreviations**
ASA: American Society of Anesthesiologists
eGFR: estimated glomerular filtration rate
GFR: glomerular filtration rate
PN: partial nephrectomy
RAPN: robot‐assisted partial nephrectomy
RN: radical nephrectomy

## 1 Introduction

Currently, surgery remains the only curative approach for treating localized renal cell cancer. In terms of preservation of renal function, partial nephrectomy (PN) rather than radical nephrectomy (RN) should be the first choice for localized T1 renal cell carcinoma, irrespective of the surgical approach [1, 2]. Robot‐assisted PN (RAPN) was first reported by Gettman et al. in 2004 [3] and has gradually gained popularity since its introduction into clinical practice. Multiple studies have examined the perioperative outcomes of RAPN and reported that this procedure can achieve results comparable to or superior to those obtained using open and laparoscopic PN [4, 5]. The indications for RAPN are expanding, and when performed by specialists, it offers a secure and viable treatment option for RENAL score 10–12 tumors [6], which are classified as highly complex [7, 8]. Regardless of the method of surgery, the aim of PN is to ensure complete tumor resection with negative surgical margins, avoid complications, and preserve renal function [9]. The preserved parenchymal volume percentage is considered the most significant predictor of preserved ipsilateral glomerular filtration rate (GFR) [10]. Therefore, current guidelines do not offer recommendations regarding the reconstruction technique during PN for achieving optimal outcomes [1, 2].

Parenchymal renorrhaphy is widely used to avoid postoperative complications by ensuring hemostasis and urinary tract closure. However, it has been reported that damage to the vascular parenchyma caused by excessive parenchymal renorrhaphy can affect postoperative renal function [11, 12]. RENAL score 10–12 tumors are large and often have an endophytic morphology or hilar location. This results in a larger reconstruction area and exposure of the renal sinus, where the large vessels and collecting system are located, leading to a larger renorrhaphy field and decreased renal blood flow. Several recent studies have assessed the safety and effectiveness of the non‐renorrhaphy technique during RAPN [13, 14, 15]. Furthermore, Batler et al. reported that the non‐renorrhaphy technique leads to renal volume preservation [16]. However, high tumor complexity may affect the surgical outcomes [17]. Only a few reports have focused on the efficacy and safety of the non‐renorrhaphy technique during RAPN for RENAL score 10–12 tumors. In this study, we aimed to retrospectively analyze the surgical outcomes of the non‐renorrhaphy technique during RAPN performed by a single surgeon for RENAL score 10–12 tumors. Additionally, we also aimed to assess the effect of this approach on renal volume preservation.

## 2 Methods

### 2.1 Study Population

Following institutional review board approval, consecutive patients with clinically localized kidney cancer treated with RAPN at our institution from March 2015 to December 2023 were retrospectively reviewed. The analysis included 51 patients with unilateral RENAL score 10–12 tumors without metastasis. Hilar involvement was observed in seven patients (44%) in the nonrenorrhaphy group and 19 patients (54%) in the renorrhaphy group. Completely endophytic features were present in nine patients (56%) in the nonrenorrhaphy group and 13 patients (37%) in the renorrhaphy group. All the surgeries included in this study were performed by a single skilled surgeon. For each patient, the following variables were evaluated: age, sex, body mass index, presence of diabetes mellitus and hypertension, American Society of Anesthesiologists (ASA) score, RENAL score, clinical and pathological tumor size, presence of hilar tumor, presence of endophytic tumor, operative time, estimated blood loss, blood transfusion, warm ischemia time, renal parenchymal volume preservation rate, preoperative and postoperative estimated GFR (eGFR) [18], other pathological factors (pT stage, histology, and positive surgical margins), perioperative complications, and recurrence. Perioperative complications were graded according to the Clavien‐Dindo classification [19].

### 2.2 Surgical Technique

The da Vinci Si, X, and Xi systems (Intuitive Surgical, Sunnyvale, CA, USA) were used to perform RAPN for all patients. Laparoscopic bulldog clamps were used to completely clamp the main renal artery. If the surgery was performed on the right side, additional clamping for the venous system was performed as needed. Enucleation or enucleoresection [20] was performed to ensure tumor resection with minimal margins. After tumor resection, a medial running suture (3‐0 Monocryl suture) was applied only to the open urinary collection system or bleeding vessel stump. The renal artery was unclamped as soon as the inner suturing was completed. If no significant arterial or venous bleeding was observed, cortical renorrhaphy was omitted, and a TachoSil patch was applied to the resection surface for hemostasis (non‐renorrhaphy technique group). In cases where hemostasis was challenging, cortical renorrhaphy was performed using barbed sutures (renorrhaphy technique group). Soft coagulation was not used in either group because of its potential impact on renal function caused by thermal injury.

### 2.3 Renal Functional Evaluation and Volumetric Analysis

Renal function assessments were conducted within 2 months prior to the surgery and again 1 month postoperatively. Enhanced computed tomography scans, used for volumetric analysis, were performed within 2 months before the surgery and 3 months after the surgery. Using SYNAPSE VINCENT (Fujifilm, Tokyo, Japan), the total renal parenchyma volume of the operated kidney was estimated from axial venous phase scans obtained at 5‐mm intervals. Regions of interest were defined at each 5‐mm slice and summed using a freehand script to determine the total renal parenchymal volume. Cysts, renal sinus fat, and the collecting system were not included in the analysis. In patients with renal dysfunction or a history of asthma, magnetic resonance imaging or planar computed tomography was used as an alternative imaging modality.

### 2.4 Statistical Analysis

We used chi‐square, Mann–Whitney *U*, and Kruskal–Wallis tests for continuous and categorical variables, as appropriate. Univariate and multivariate linear regression techniques were employed to identify variables independently associated with the preservation rate of renal parenchymal volume and eGFR. A *p* value < 0.05 was considered statistically significant. All statistical analyses were performed using the JMP 13.0 software (SAS Institute, Cary, NC).

## 3 Results

Patient and tumor characteristics are shown in Table 1. Altogether, 51 patients were included in the analysis, comprising 16 patients in the nonrenorrhaphy group and 35 in the renorrhaphy group. The median age at the time of surgery was 56 years, and 73% of the patients were men. Median body mass index was 24.8 kg/m^2^; 12% of patients had diabetes as a comorbidity, and 43% had hypertension. ASA distribution was as follows: ASA 1, 12%; ASA 2, 67%; and ASA 3, 21%. The median clinical tumor size was 45 mm, with no case of cT2 or larger tumors. Hilar tumors constituted 51%, and completely endophytic tumors accounted for 43%. No statistically significant difference was observed in the baseline characteristics between the two groups.

**TABLE 1:** Patient and tumor characteristics.

Factors	Nonrenorrhaphy	Renorrhaphy	Total	*p*
Number, *n*	16	35	51	
Sex, male, *n* (%)	10 (63)	27 (77)	37 (73)	0.2769
Age, year, median (IQR)	62 (54–72)	51 (44–69)	56 (46–70)	0.0603
BMI, kg/m^2^, median (IQR)	25.3 (22.6–27.2)	24.3 (20.1–27.5)	24.8 (21.6–27.3)	0.4110
DM, *n* (%)	2 (13)	4 (11)	6 (12)	0.9123
HTN, *n* (%)	8 (50)	14 (40)	22 (43)	0.5035
Preoperative eGFR, mL/min/1.73 m^2^, median (IQR)	69.7 (54.1–80.5)	67.8 (53.2–78)	67.9 (53.2–78.3)	0.7073
Chronic kidney disease > stage 3, *n* (%)	4 (25)	11 (31)	15 (29)	0.6401
ASA score, *n* (%)				
1	1 (6)	5 (14)	6 (12)	0.4325
2	10 (63)	24 (69)	34 (67)	
3	5 (31)	6 (17)	11 (21)	
RENAL nephrometry score, *n* (%)				
10	16 (100)	34 (97)	50 (98)	0.4947
11	0 (0)	1 (3)	1 (2)	
Clinical tumor size (mm), median (IQR)	39.5 (25.75–47.5)	46 (40–60)	45 (36–60)	0.0721
Clinical stage, *n* (%)				
cT1a	8 (50)	9 (26)	16 (31)	0.0878
cT1b	8 (50)	26 (67)	35 (69)	
> cT2a	0 (0)	0 (0)	0 (0)	
Hilar tumor, *n* (%)	7 (44)	19 (54)	26 (51)	0.4849
Completely endophytic tumor, *n* (%)	9 (56)	13 (37)	22 (43)	0.2011

Abbreviations: ASA, American Society of Anesthesiologists; BMI, body mass index; DM, diabetes mellitus; eGFR, estimated glomerular filtration rate; HTN, hypertension; IQR, interquartile range.

Table 2 presents the pathological findings. Malignant tumors were 14 (88%) in the nonrenorrhaphy group and 31 (89%) in the renorrhaphy group. Upstaging to pT3a was not observed. No significant differences were found in pathological stage or histology between the two groups. In one case in the nonrenorrhaphy group, the pathology specimen was poorly fixed, resulting in a pTx assessment because the surgical margins could not be accurately evaluated. The positive surgical margin rate was 0% in the nonrenorrhaphy group versus 10% in the renorrhaphy group (*p* = 0.4849).

**TABLE 2:** Pathological reports.

Factors	Nonrenorrhaphy	Renorrhaphy	Total	*p*
Pathological tumor size, median, mm, (IQR)	33 (18–44)	50 (31–55)	42 (30–55)	0.0337
Pathological stage, *n* (%)				
Benign	2 (13)	4 (11)	6 (12)	0.0581
T1a	9 (56)	10 (29)	19 (37)	
T1b	4 (15)	19 (54)	23 (45)	
T2a	0 (0)	2 (6)	2 (4)	
T3a	0 (0)	0 (0)	0 (0)	
Tx	1 (6)	0 (0)	1 (2)	
Histology, *n* (%)				
Clear cell RCC	11 (69)	26 (74)	37 (73)	0.9483
Chromophobe RCC	2 (13)	4 (11)	6 (12)	
Papillary RCC	1 (6)	1 (3)	2 (4)	
Benign tumor	2 (13)	4 (11)	6 (12)	
Positive surgical margin, *n* (%)	0 (0)	3 (9)	3 (6)	0.4849

Abbreviations: IQR, interquartile range; RCC, renal cell carcinoma.

Table 3 summarizes the comparisons of surgical outcomes, renal function, and parenchymal volume between the two groups. The nonrenorrhaphy group had a significantly shorter operative time than the renorrhaphy group (185 vs. 217 min, *p* = 0.0016). The median duration of cortical renorrhaphy in the renorrhaphy group was 9 min. The preservation rate of renal parenchymal volume was significantly higher in the nonrenorrhaphy group than in the renorrhaphy group (86.7 vs. 74.2%, *p* = 0.0016), whereas no significant differences were observed in the preservation rate of eGFR (*p* = 0.6380), warm ischemia time (*p* = 0.2550), and estimated blood loss (*p* = 0.7040). The incidence of complications was not significantly different between the groups (nonrenorrhaphy: one case, nonrenorrhaphy: three cases, *p* = 0.7748). Collecting system entry was observed during tumor resection in all cases included in this study. Urinary leakage occurred in one patient in the nonrenorrhaphy group and two patients in the renorrhaphy group; however, there was no statistically significant difference between the groups (*p* = 0.9399). In the nonrenorrhaphy group, one patient developed a urinoma requiring readmission. Urinary leakage did not improve with ureteral stenting and drainage but was ultimately resolved after fibrin infusion. The preservation rate of eGFR was 90% at 1 month after resolution and 79% at 4 years postoperatively.

**TABLE 3:** Comparison of surgical outcomes.

Factors	Nonrenorrhaphy	Renorrhaphy	Total	*p*
OT, min, median (IQR)	185 (170–198)	217 (197–236)	206 (184–233)	**0.0016**
Cortical renorrhaphy time, min, median (IQR)	—	9 (7–12)	—	—
EBL, mL, median (IQR)	100 (50–115)	100 (50–160)	100 (50–150)	0.7040
Transfusion, *n* (%)	1 (6)	0 (0)	1 (2)	0.1352
WIT, min, median (IQR)	25 (22–32)	27 (21–36)	27 (22–34)	0.2550
Preservation rate of renal parenchymal volume, %, median (IQR)	86.7 (76.1–94.1)	74.2 (68.3–82.3)	76.1 (71.3–85.3)	**0.0016**
Preop eGFR, mL/min/1.73 m^2^, median (IQR)	69.7 (54.1–80.5)	67.8 (53.2–78)	67.9 (53.2–78.3)	0.7073
Postop eGFR, mL/min/1.73 m^2^, median (IQR)	67.3 (49.0–74.1)	63.4 (54.5–69.2)	63.97 (71.9–54.0)	0.5534
Preservation rate of eGFR, %, median (IQR)	90.1 (86.7–98.7)	89.4 (83.2–95.9)	89.4 (84.8–95.9)	0.6380
Collecting system entry, *n* (%)	16 (100)	35 (100)	51 (100)	—
Complications, *n* (Clavien‐Dindo ≥ G3)	1 (6)	3 (9)	4 (8)	0.7748
Urinary leakage, *n* (Clavien‐Dindo ≥ G3)	1 (6)	2 (6)	3 (6)	0.9399
RAP, *n* (Clavien‐Dindo ≥ G3)	0 (0)	0 (0)	0 (0)	—
Conversion to radical nephrectomy, *n* (%)	0 (0)	0 (0)	0 (0)	—
Local recurrence, *n*	0 (0)	1 (3)	1 (2)	0.3293
Metastatic recurrence, *n*	1 (6)	4 (11)	5 (11)	0.5639
Median observation period, month (IQR)	20.5 (15.25–36.5)	49 (28–69)	36 (19–64)	**0.0025**

*Note:* Bold values indicate that the comparison between the two groups was considered statistically significant when the *p*‐value was less than 0.05.

Abbreviations: EBL, estimated blood loss; eGFR, estimated glomerular filtration rate; IQR, interquartile range; OT, operative time; Postop, postoperative; Preop, preoperative; RAP, renal artery pseudoaneurysm; WIT, warm ischemia time.

In the renorrhaphy group, one patient required readmission for urinary leakage caused by injury to the ureteropelvic junction during tumor exposure. Despite attempted management with ureteral stenting and drainage, RN was eventually performed because of poor vascularity and persistent leakage. Another patient developed urinary leakage during hospitalization, which was successfully managed with ureteral stenting alone. The patient's eGFR preservation was 79% at 1 month and 87% at 1 year posttreatment.

No renal artery pseudoaneurysm was observed in the two groups. Local recurrence was observed in one case, and distant metastasis in five cases altogether. The recurrence rate did not differ between the two groups. The median follow‐up duration was 36 months.

Univariate and multivariate linear regression analyses of predictive factors for preservation of renal parenchymal volume and eGFR—including age, sex, clinical tumor size, warm ischemia time, and renorrhaphy—are presented in Table 4. One patient who underwent nephrectomy because of urinary leakage and three patients who underwent postoperative ultrasound follow‐up for benign tumors were excluded from this analysis. In univariate and multivariate analyses, preoperative tumor size and renorrhaphy were statistically significant predictors of preservation of parenchymal volume (univariate analysis: *p* = 0.00781 and 0.00144; multivariate analysis: *p* = 0.0343 and 0.0127, respectively). No significant predictive factors for eGFR preservation were identified in either analysis.

**TABLE 4:** Univariate and multivariate regression analysis on preservation of renal parenchymal volume and eGFR.

	Univariate analysis		Multivariate analysis	
	Co‐efficient *β* (95% CI)	*p*	Co‐efficient *β* (95% CI)	*p*
Preservation of renal parenchymal volume				
Age	0.158 (−0.0606, 0.376)	0.153	0.120 (−0.0921, 0.333)	0.260
Sex	0.714 (−6.62, 8.05)	0.845	3.50 (−3.10, 10.1)	0.290
Tumor size	−0.233 (−0.401, −0.0644)	**0.00781**	−0.182 (−0.351, −0.0142)	**0.0343**
WIT	−0.249 (−0.610, 0.113)	0.173	−0.000753 (−0.356, 0.355)	0.997
Renorrhaphy	−10.5 (−16.8, −4.28)	**0.00144**	−8.42 (−15.0, −1.89)	**0.0127**
Preservation of eGFR				
Age	−0.0427 (−0.206, 0.120)	0.601	−0.0969 (−0.274, 0.0800)	0.276
Sex	−1.41 (−6.77, 3.96)	0.601	−1.12 (−6.58, 4.35)	0.683
Tumor size	−0.0764 (−0.216, 0.0630)	0.276	−0.0452 (−0.195, 0.105)	0.548
WIT	−0.214 (−0.490, 0.0611)	0.124	−0.237 (−0.549, 0.0756)	0.134
Renorrhaphy	−1.24 (−6.41, 3.92)	0.631	−0.385 (−5.96, 5.19)	0.890

*Note:* Bold values indicate that the comparison between the two groups was considered statistically significant when the *p*‐value was less than 0.05.

Abbreviations: CI, confidence interval; eGFR, estimated glomerular filtration rate; WIT, warm ischemia time.

## 4 Discussion

In this study, we assessed the feasibility and safety of the non‐renorrhaphy technique in RAPN for RENAL score 10–12 tumors and evaluated its contribution to renal volume preservation. Three major findings were highlighted in this study. First, the non‐renorrhaphy technique significantly reduced operative time, with a median time of 185 min, in contrast to 217 min in the renorrhaphy group (*p* = 0.0016). However, no significant differences were observed in warm ischemia time or estimated blood loss between the two groups (*p* = 0.2550 and 0.7040, respectively; Table 3). The shorter operative time in the nonrenorrhaphy group is likely because of the omission of the cortical renorrhaphy step, which required a median of 9 min (range: 7–12 min) in the renorrhaphy group. Furthermore, the renorrhaphy group included more cases with larger tumors, which may have prolonged the time required for dissection prior to tumor excision.

Second, no significant difference was observed in the incidence of major complications (Clavien‐Dindo grade ≥ 3) between the two groups. No hemorrhagic complications, including renal artery pseudoaneurysm, were observed in either group. Urinary leakage occurred in one case (6%) in the nonrenorrhaphy group and two cases (6%) in the renorrhaphy group (Table 3). Third, non‐renorrhaphy technique and tumor size were predictors of renal parenchyma preservation. However, no significant predictors of eGFR preservation were found in this analysis (Table 4).

Our findings agree with previous studies demonstrating the benefits of the non‐renorrhaphy technique in renal volume preservation. For example, Bahler et al. showed that the percentage of volume loss is significantly lower in the nonrenorrhaphy group, at 9.0% compared to 17.0% in the renorrhaphy group (*p* = 0.003) [16]. This volume preservation is presumed to result from avoidance of the parenchymal hypoperfusion or strangulation caused by renorrhaphy suturing. However, they observed no statistically significant differences in terms of %GFR loss between the two groups. Moreover, several studies have suggested that the non‐renorrhaphy technique during RAPN is not significantly associated with impaired renal function [14, 15]. In fact, a systematic review indicated that nonrenorrhaphy may have some renal functional benefits, though the evidence remains limited because of the small number of studies included [21]. Parenchymal hypoperfusion and strangulation resulting from cortical renorrhaphy are not the only factors associated with reconstruction‐induced volume loss and recovery of kidney function after PN. Following nephrectomy, compensatory structural hypertrophy and functional hyperfiltration occur in the remnant renal parenchyma as physiological adaptations aimed at preserving renal function. These mechanisms may have contributed to the absence of a significant difference in eGFR preservation observed in this study. We evaluated only the volume of the operated kidney and global renal function, without directly assessing functional changes in the ipsilateral kidney. To more accurately assess the impact of renal parenchymal volume preservation achieved by the non‐renorrhaphy technique, future studies should incorporate functional evaluation of the ipsilateral kidney using modalities such as renal scintigraphy. Moreover, it is not yet clear what the long‐term effects are of inducing parenchymal strangulation or hypoperfusion. To better understand the long‐term renal functional outcomes of this approach, larger‐scale studies are required.

Several previous reports have suggested no differences in postoperative major complications between renorrhaphy and non‐renorrhaphy techniques during RAPN [14, 15, 16]. Our findings were consistent with those of previous studies. We did not observe hemorrhagic complications, including renal artery pseudoaneurysm, in either of the two groups. Renal artery pseudoaneurysm is one of the fatal complications of PN. Injury to the renal artery caused by large‐needle suturing during renorrhaphy or failure to achieve adequate hemostasis of arterial bleeding from the resected tumor bed may result in the formation of a renal artery pseudoaneurysm [22]. After PN for RENAL score 10–12 tumors, the tumor base often exposes the renal sinus, which contains the segmental arteries. Consequently, blind renorrhaphy with large‐needle sutures poses a greater risk of injuring major blood vessels. The non‐renorrhaphy technique may help reduce the risk of hemorrhagic complications, including renal artery pseudoaneurysm, especially in RENAL score 10–12 cases. However, Batler et al. recommended monitoring the tumor resection bed for at least 20 min after arterial clamping, as vasospasm may obscure bleeding [16].

The effectiveness of renorrhaphy in preventing urinary leakage has been reported. In a study on open PN, Tachibana et al. reported that the incidence of urinary leakage was significantly lower in the renorrhaphy group (2.3%) compared to the nonrenorrhaphy group (20%), supporting the preventive role of the renorrhaphy technique [23]. Conversely, the incidence of urinary leakage in RAPN was very low (0.78%), likely because of enhanced visualization and improved suturing techniques afforded by the robotic approach [24]. Although limited in number, previous studies reporting non‐renorrhaphy techniques in RAPN did not report any cases of postoperative urinary leakage [13, 14, 16]. However, in our present study, urinary leakage occurred in both groups.

Stroup et al. identified higher RENAL scores as a risk factor for urinary leakage [25]. Potretzke et al. also reported that tumor complexity, including factors such as tumor size and hilar location, was significantly associated with the occurrence of urinary leakage [24]. All tumors included in this study had RENAL scores of 10–12. Hilar tumors accounted for 51%, and collecting system entry was observed in all cases. These tumor characteristics may have contributed to an increased risk of urinary leakage in this study.

Nevertheless, renorrhaphy was not significantly associated with urinary leakage (*p* = 0.7748). Because of the limited sample size, these results may be subject to bias. This finding may suggest that secure closure of the urinary tract, rather than the renorrhaphy technique itself, is more important in preventing urinary leakage. However, as the resection area increases, complete closure of the urinary tract becomes more difficult; therefore, performing renorrhaphy may be safer in such cases.

This study had some limitations. First, the retrospective design and the fact that the study was conducted at a single center may limit the ability to generalize the findings. The small sample size made it difficult to balance baseline characteristics between the two groups, which may have influenced the results. Additionally, the choice of surgical technique was not preoperatively planned but determined intraoperatively based on hemostatic status after tumor resection, introducing the possibility of selection bias. Furthermore, since this study was conducted by a single surgeon, the potential impact of the surgeon's learning curve on the outcomes should be considered. The surgeon introduced the non‐renorrhaphy technique after performing over 200 RAPN cases using renorrhaphy. The outcomes reflect procedures performed by an expert with extensive experience in the standard technique and may not be directly generalizable to other institutions or surgeons with different levels of RAPN experience. Finally, patients with tumor diameter ≥ 70 mm could not be included in this study because of the indications for RAPN in our country.

Despite these limitations, the strengths of this study lie in demonstrating the feasibility and safety of the non‐renorrhaphy technique in RAPN for RENAL score 10–12 tumors. Our findings indicate that the non‐renorrhaphy technique may be a viable option for surgeons aiming to preserve renal parenchyma while reducing operative time, without compromising surgical outcomes. We believe that this report is relevant to real‐world clinical practice. Although it is important to report actual clinical outcomes using the non‐renorrhaphy technique, additional multicenter, prospective studies with larger sample sizes and longer follow‐up are necessary to verify these findings and evaluate the long‐term effects on renal function.

## Author Contributions

**Kosuke Hamada:** conceptualization, writing – original draft, data curation, investigation, methodology, formal analysis, visualization, validation, writing – review and editing. **Takeshi Yamasaki:** conceptualization, writing – review and editing. **Shoma Yamamoto:** investigation. **Taisuke Matsue:** investigation. **Nao Yukimatsu:** investigation. **Taiyo Otoshi:** validation. **Minoru Kato:** validation. **Katsuyuki Kuratsukuri:** validation. **Junji Uchida:** supervision.

## Ethics Statement

This study was approved by the institutional ethics committee at Osaka Metropolitan University (approval number 2022‐050), and it conforms to the provisions of the Declaration of Helsinki.

## Consent

Before the surgery, all patients provided written informed consent for RAPN.

## Conflicts of Interest

Junji Uchida is an Editorial Board member of the *International Journal of Urology* and a co‐author of this article. To minimize bias, they were excluded from all editorial decision‐making related to the acceptance of this article for publication.

## References

[1] S. C. Campbell , P. E. Clark , S. S. Chang , J. A. Karam , L. Souter , and R. G. Uzzo , “Renal Mass and Localized Renal Cancer: Evaluation, Management, and Follow‐Up: AUA Guideline: Part I: AUA Guideline,” Journal of Urology 206, no. 2 (2021): 199–208. 34115547 10.1097/JU.0000000000001911

[2] B. Ljungberg , L. Albiges , Y. Abu‐Ghanem , et al., “European Association of Urology Guidelines on Renal Cell Carcinoma: The 2022 Update,” European Urology 82, no. 4 (2022): 399–410. 35346519 10.1016/j.eururo.2022.03.006

[3] M. T. Gettman , M. L. Blute , G. K. Chow , R. Neururer , G. Bartsch , and R. Peschel , “Robotic‐Assisted Laparoscopic Partial Nephrectomy: Technique and Initial Clinical Experience With DaVinci Robotic System,” Urology 64, no. 5 (2004): 914–918. 15533477 10.1016/j.urology.2004.06.049

[4] J. J. Leow , N. H. Heah , S. L. Chang , Y. L. Chong , and K. S. Png , “Outcomes of Robotic Versus Laparoscopic Partial Nephrectomy: An Updated Meta‐Analysis of 4,919 Patients,” Journal of Urology 196, no. 5 (2016): 1371–1377. 27291654 10.1016/j.juro.2016.06.011

[5] J. Simhan , M. C. Smaldone , K. J. Tsai , et al., “Perioperative Outcomes of Robotic and Open Partial Nephrectomy for Moderately and Highly Complex Renal Lesions,” Journal of Urology 187, no. 6 (2012): 2000–2004. 22498208 10.1016/j.juro.2012.01.064

[6] A. Kutikov and R. G. Uzzo , “The R.E.N.A.L. Nephrometry Score: A Comprehensive Standardized System for Quantitating Renal Tumor Size, Location and Depth,” Journal of Urology 182, no. 3 (2009): 844–853. 19616235 10.1016/j.juro.2009.05.035

[7] D. B. Hennessey , G. Wei , D. Moon , et al., “Strategies for Success: A Multi‐Institutional Study on Robot‐Assisted Partial Nephrectomy for Complex Renal Lesions,” BJU International 121, no. Suppl 3 (2018): 40–47. 29072806 10.1111/bju.14059

[8] N. M. Buffi , A. Saita , G. Lughezzani , et al., “Robot‐Assisted Partial Nephrectomy for Complex (PADUA Score >/=10) Tumors: Techniques and Results From a Multicenter Experience at Four High‐Volume Centers,” European Urology 77, no. 1 (2020): 95–100. 30898407 10.1016/j.eururo.2019.03.006

[9] A. J. Hung , J. Cai , M. N. Simmons , and I. S. Gill , ““Trifecta” in Partial Nephrectomy. Trifecta,” Journal of Urology 189, no. 1 (2013): 36–42. 23164381 10.1016/j.juro.2012.09.042

[10] A. Kazama , W. Attawettayanon , C. Munoz‐Lopez , et al., “Parenchymal Volume Preservation During Partial Nephrectomy: Improved Methodology to Assess Impact and Predictive Factors,” BJU International 134, no. 2 (2024): 219–228. 38355293 10.1111/bju.16300

[11] C. D. Bahler and C. P. Sundaram , “Effect of Renal Reconstruction on Renal Function After Partial Nephrectomy,” Journal of Endourology 30, no. Suppl 1 (2016): S37–S41. 26864480 10.1089/end.2016.0055

[12] F. Porpiglia , R. Bertolo , D. Amparore , and C. Fiori , “Nephron‐Sparing Suture of Renal Parenchyma After Partial Nephrectomy: Which Technique to Go for? Some Best Practices,” European Urology Focus 5, no. 4 (2019): 600–603. 28869203 10.1016/j.euf.2017.08.006

[13] Y. Tohi , S. Murata , N. Makita , et al., “Comparison of Perioperative Outcomes of Robot‐Assisted Partial Nephrectomy Without Renorrhaphy: Comparative Outcomes of cT1a Versus cT1b Renal Tumors,” International Journal of Urology 26, no. 9 (2019): 885–889. 31257682 10.1111/iju.14046

[14] I. Alrishan Alzouebi , A. Williams , N. R. Thiagarjan , and M. Kumar , “Omitting Cortical Renorrhaphy in Robot‐Assisted Partial Nephrectomy: Is It Safe? A Single Center Large Case Series,” Journal of Endourology 34, no. 8 (2020): 840–846. 32316759 10.1089/end.2020.0121

[15] S. Arora , C. Bronkema , J. R. Porter , et al., “Omission of Cortical Renorrhaphy During Robotic Partial Nephrectomy: A Vattikuti Collective Quality Initiative Database Analysis,” Urology 146 (2020): 125–132. 32941944 10.1016/j.urology.2020.09.003

[16] C. D. Bahler , H. T. Dube , K. J. Flynn , et al., “Feasibility of Omitting Cortical Renorrhaphy During Robot‐Assisted Partial Nephrectomy: A Matched Analysis,” Journal of Endourology 29, no. 5 (2015): 548–555. 25616087 10.1089/end.2014.0763

[17] M. H. Hayn , T. Schwaab , W. Underwood , and H. L. Kim , “RENAL Nephrometry Score Predicts Surgical Outcomes of Laparoscopic Partial Nephrectomy,” BJU International 108, no. 6 (2011): 876–881. 21166761 10.1111/j.1464-410X.2010.09940.x

[18] S. Matsuo , E. Imai , M. Horio , et al., “Revised Equations for Estimated GFR From Serum Creatinine in Japan,” American Journal of Kidney Diseases 53, no. 6 (2009): 982–992. 19339088 10.1053/j.ajkd.2008.12.034

[19] D. Dindo , N. Demartines , and P. A. Clavien , “Classification of Surgical Complications: A New Proposal With Evaluation in a Cohort of 6336 Patients and Results of a Survey,” Annals of Surgery 240, no. 2 (2004): 205–213. 15273542 10.1097/01.sla.0000133083.54934.ae PMC1360123

[20] A. Minervini , M. Carini , R. G. Uzzo , R. Campi , M. C. Smaldone , and A. Kutikov , “Standardized Reporting of Resection Technique During Nephron‐Sparing Surgery: The Surface‐Intermediate‐Base Margin Score,” European Urology 66, no. 5 (2014): 803–805. 24954792 10.1016/j.eururo.2014.06.002

[21] R. Bertolo , R. Campi , M. C. Mir , et al., “Systematic Review and Pooled Analysis of the Impact of Renorrhaphy Techniques on Renal Functional Outcome After Partial Nephrectomy,” European Urology Oncology 2, no. 5 (2019): 572–575. 31412012 10.1016/j.euo.2018.11.008

[22] T. Kondo , T. Takagi , S. Morita , et al., “Early Unclamping Might Reduce the Risk of Renal Artery Pseudoaneurysm After Robot‐Assisted Laparoscopic Partial Nephrectomy,” International Journal of Urology 22, no. 12 (2015): 1096–1102. 26307333 10.1111/iju.12902

[23] H. Tachibana , T. Takagi , T. Kondo , H. Ishida , and K. Tanabe , “Comparison of Perioperative Outcomes With or Without Renorrhaphy During Open Partial Nephrectomy: A Propensity Score‐Matched Analysis,” International Brazilian Journal of Urology 44, no. 3 (2018): 467–474. 29244272 10.1590/S1677-5538.IBJU.2016.0581 PMC5996815

[24] A. M. Potretzke , B. A. Knight , H. Zargar , et al., “Urinary Fistula After Robot‐Assisted Partial Nephrectomy: A Multicentre Analysis of 1791 Patients,” BJU International 117, no. 1 (2016): 131–137. 26235802 10.1111/bju.13249

[25] S. P. Stroup , K. Palazzi , R. P. Kopp , et al., “RENAL Nephrometry Score Is Associated With Operative Approach for Partial Nephrectomy and Urine Leak,” Urology 80, no. 1 (2012): 151–156. 22748871 10.1016/j.urology.2012.04.026

